# Targeted Vagus Nerve Stimulation for Rehabilitation After Stroke

**DOI:** 10.3389/fnins.2019.00280

**Published:** 2019-03-29

**Authors:** Navzer D. Engineer, Teresa J. Kimberley, Cecília N. Prudente, Jesse Dawson, W. Brent Tarver, Seth A. Hays

**Affiliations:** ^1^MicroTransponder, Inc., Austin, TX, United States; ^2^Department of Physical Therapy, School of Health and Rehabilitation Sciences, MGH Institute of Health Professions, Boston, MA, United States; ^3^Institute of Cardiovascular and Medical Sciences, College of Medical, Veterinary and Life Sciences, Queen Elizabeth University Hospital, University of Glasgow, Glasgow, United Kingdom; ^4^Texas Biomedical Device Center, The University of Texas at Dallas, Richardson, TX, United States; ^5^Department of Bioengineering, The University of Texas at Dallas, Richardson, TX, United States

**Keywords:** stroke, vagus nerve stimulation, plasticity, rehabilitation, neuromodulation

## Abstract

Stroke is a leading cause of disability worldwide, and in approximately 60% of individuals, upper limb deficits persist 6 months after stroke. These deficits adversely affect the functional use of the upper limb and restrict participation in day to day activities. An important goal of stroke rehabilitation is to improve the quality of life by enhancing functional independence and participation in activities. Since upper limb deficits are one of the best predictors of quality of life after stroke, effective interventions targeting these deficits may represent a means to improve quality of life. An increased understanding of the neurobiological processes underlying stroke recovery has led to the development of targeted approaches to improve motor deficits. One such targeted strategy uses brief bursts of Vagus Nerve Stimulation (VNS) paired with rehabilitation to enhance plasticity and support recovery of upper limb function after chronic stroke. Stimulation of the vagus nerve triggers release of plasticity promoting neuromodulators, such as acetylcholine and norepinephrine, throughout the cortex. Timed engagement of neuromodulators concurrent with motor training drives task-specific plasticity in the motor cortex to improve function and provides the basis for paired VNS therapy. A number of studies in preclinical models of ischemic stroke demonstrated that VNS paired with rehabilitative training significantly improved the recovery of forelimb motor function compared to rehabilitative training without VNS. The improvements were associated with synaptic reorganization of cortical motor networks and recruitment of residual motor neurons controlling the impaired forelimb, demonstrating the putative neurobiological mechanisms underlying recovery of motor function. These preclinical studies provided the basis for conducting two multi-site, randomized controlled pilot trials in individuals with moderate to severe upper limb weakness after chronic ischemic stroke. In both studies, VNS paired with rehabilitation improved motor deficits compared to rehabilitation alone. The trials provided support for a 120-patient pivotal study designed to evaluate the efficacy of paired VNS therapy in individuals with chronic ischemic stroke. This manuscript will discuss the neurobiological rationale for VNS therapy, provide an in-depth discussion of both animal and human studies of VNS therapy for stroke, and outline the challenges and opportunities for the future use of VNS therapy.

## Introduction

Stroke is a leading cause of disability and a significant health burden in the United States and worldwide (Murray et al., 2013; Feigin et al., 2016). Upper limb deficits persist in approximately 60% of individuals after stroke (Wade et al., 1983), limiting their use in day to day activities and impacting quality of life of the individual (Franceschini et al., 2010; Morris et al., 2013). An important goal of stroke rehabilitation research is to develop effective, evidence-based therapies to reduce impairment, facilitate functional upper limb use and improve participation in activities without resorting to compensatory strategies after chronic stroke.

Neurophysiological and neuroimaging studies have provided an improved understanding of the neurobiological processes underlying the brain’s ability to restore function by capitalizing on residual networks after stroke (Krakauer, 2004; Ward, 2004; Nudo, 2006; Murphy and Corbett, 2009; Dimyan and Cohen, 2011; Boyd et al., 2017; Sampaio-Baptista et al., 2018). One approach for improving chronic upper limb deficits is to augment this capacity to reorganize, referred to as plasticity. Rehabilitation by itself drives some reorganization of motor networks, but these changes occur within a framework of architectural and anatomical constraints which are believed to limit substantial improvements (Kleim and Jones, 2008). As a result, strategies that can enhance reorganization in conjunction with rehabilitation may support greater recovery. Here, we will describe the neurophysiological basis and implementation of VNS during rehabilitation as a means to enhance plasticity and improve post-stroke recovery.

## Cholinergic and Noradrenergic Modulation of Cortical Plasticity

Activation of neuromodulatory networks is strongly linked to plasticity (Gu, 2002), thus engaging these mechanisms provides a potential strategy to enhance plasticity for stroke recovery. Cholinergic neurons within the nucleus basalis (NB) and noradrenergic neurons in the locus coeruleus (LC) are part of the ascending neuromodulatory system that projects diffusely to wide areas of the cortex. Release of acetylcholine (ACh) from NB neurons and norepinephrine (NE) from LC neurons plays an important role in many behavioral and cognitive processes including arousal, memory consolidation and attentional modulation of goal-directed behavior (Gu, 2002; Aston-Jones and Cohen, 2005; Sarter et al., 2005; Hasselmo and Sarter, 2011). The vagus nerve sends projections to the nucleus tractus solitarius (NTS), which in turn projects to the neuromodulatory nuclei. Therefore, understanding the role of these neuromodulatory networks in cortical plasticity is instructive for defining the basis for delivering VNS paired with sensory or motor events to facilitate plasticity.

In a constantly changing world, the brain must extract behaviorally relevant information to drive useful goal-directed behaviors. Neuromodulatory networks, including the cholinergic and noradrenergic systems which provide diffuse neuromodulatory innervation throughout the cortex, are uniquely poised to serve that role. Cholinergic and noradrenergic neurons show phasic discharge during specific epochs of behavior that may signal cue detection, novelty or reinforcement feedback (Hasselmo, 1995; Arnold et al., 2002; Bouret and Sara, 2004; Sarter et al., 2005, 2006, 2009; Parikh et al., 2007; Hasselmo and Sarter, 2011). For example, transient cholinergic activity in cortical neurons signals behaviorally relevant cues while decreased activity is observed with missed cues (Parikh et al., 2007). Rapid cholinergic activation provides reinforcement feedback in response to both positive and negative events (Hangya et al., 2015). Similarly, phasic discharge from LC neurons predicts correct responses in a visual discrimination task with increased cross-correlation among LC neurons (Usher et al., 1999). These studies demonstrate that brief bursts of ACh or NE are likely involved in the attentional modulation of cortical neurons to encode the behavioral relevance of stimulus-specific features during task performance.

The neuromodulator-driven attentional modulation of cortical neurons must eventually be encoded into long-lasting changes in synaptic efficacy with successful task learning (Hess and Donoghue, 1994; Hess and Krawczyk, 1996; Kirkwood et al., 1999; Rioult-Pedotti et al., 2000; Ziemann et al., 2006; Seol et al., 2007; Cohen and Maunsell, 2009; Korchounov and Ziemann, 2011; Carcea and Froemke, 2013; Hasan et al., 2013). At a systems level, the changes in synaptic efficacy may underlie reorganization of cortical maps specific to the learned features of the task (Merzenich et al., 1988; Recanzone et al., 1992, 1993; Pascual-Leone and Torres, 1993; Elbert et al., 1995; Buonomano and Merzenich, 1998; Sterr et al., 1998; Feldman and Brecht, 2005; Feldman, 2009; Froemke, 2015). Furthermore, depletion of cortical ACh or NE resulting from lesions of their respective nuclei or pharmacologic modulation with cholinergic and noradrenergic antagonists blocks cortical plasticity and impairs learning (Sato et al., 1987; Juliano et al., 1991; Heron et al., 1996; Kilgard and Merzenich, 1998; Zhu and Waite, 1998; Conner et al., 2003, 2005; Ramanathan et al., 2009; Vitrac and Benoit-Marand, 2017). Together, these studies established a causal role for the neuromodulatory networks in task-specific learning and plasticity.

The vagus nerve projects to the NTS (Foley and DuBois, 1937; Prechtl and Powley, 1990) and consequently provides rapid activation of the cholinergic and noradrenergic systems (Roosevelt et al., 2006; Nichols et al., 2011; Porter et al., 2012; Hulsey et al., 2017). Therefore, the engagement of these neuromodulatory systems by VNS led to the prediction that brief bursts of VNS paired with sensory or motor experience could enhance cortical plasticity that was specific to the paired experience. Repeatedly pairing a tone with VNS reorganized the rat auditory cortex map, resulting in an expansion for the paired tone (Engineer et al., 2011). A tone paired with trigeminal nerve stimulation did not result in specific auditory cortex plasticity, demonstrating that the enhancement of plasticity was unique to stimulation of the vagus nerve.

Neuromodulatory networks share some features in mediating plasticity in motor and auditory cortices (Gu, 2002; Ramanathan et al., 2009). Since VNS paired with sensory experience drives robust, specific plasticity in the primary sensory cortex, this raised the possibility that pairing VNS with motor training could also facilitate plasticity in naïve rat motor cortex. Indeed, repeatedly pairing VNS with a forelimb movement during motor training increased the corresponding map representation of that movement in motor cortex compared to equivalent training in rats that did not receive VNS (Porter et al., 2012). These studies laid the groundwork for using VNS paired with motor training for improving upper limb deficits after stroke.

## Vns Improves Motor Function in Animal Models of Stroke

Post-stroke recovery is associated with plasticity in motor networks (Murphy and Corbett, 2009). The development of strategies to enhance this plasticity and subsequently generate greater recovery has been the focus of intense research. Based on its ability to drive training-dependent neuroplasticity in uninjured motor networks, a number of animal studies have evaluated VNS paired with rehabilitative training to support the recovery of motor function after stroke.

A study performed in an animal model of ischemic stroke tested the hypothesis that VNS paired with rehabilitative training could enhance post-stroke recovery (Khodaparast et al., 2013). This study sought to evaluate the ability of VNS delivered during motor training to improve recovery of forelimb strength, a main contributor to disability after stroke (Canning et al., 2004; Harris and Eng, 2007). Rats were trained to proficiency on a strength-based forelimb task, and then underwent ischemic lesion of the motor cortex. Rats that received brief bursts of VNS paired with forelimb movement during motor training demonstrated significantly greater recovery of volitional forelimb strength compared to rats that received equivalent training without VNS. Recovery persisted when assessed 1 week after the cessation of stimulation, consistent with the notion that VNS drives stable plasticity and providing an initial indication that the benefits of VNS therapy may be lasting.

**FIGURE 1:**
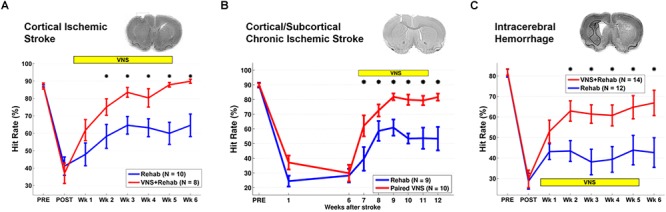
Vagus Nerve Stimulation-dependent recovery of motor function in rat models of stroke. **(A)** VNS paired with rehabilitative training significantly improves recovery of forelimb motor function compared to equivalent training without VNS in a model of cortical ischemic stroke. The top panel shows a coronal brain section with a representative ischemic lesion. Similarly, VNS paired with rehabilitative training enhances recovery of forelimb function after **(B)** chronic combined cortical and subcortical ischemic and **(C)** intracerebral hemorrhage. The symbol “^∗^” indicates *p* < 0.05 across groups at each time point (Adapted from Hays et al., 2014a,b; Khodaparast et al., 2016).

A second study built upon these initial findings and assessed the ability of VNS to improve forelimb movement speed after stroke. Rats were pre-trained on a skilled task that measured rapid movement of the forelimb and underwent an ischemic lesion of the motor cortex (Khodaparast et al., 2014). Corroborating findings from the initial study, VNS paired with forelimb movement during rehabilitative training resulted in significant enhancement of functional recovery compared to equivalent rehabilitation training without VNS (Figure 1A).

Rehabilitation can become less effective with increasing time after stroke. To evaluate whether a long delay in therapy delivery would impact the efficacy of VNS, a study evaluated whether VNS paired with rehabilitative training could improve recovery in a rat model of chronic ischemic stroke (Khodaparast et al., 2016). VNS and rehabilitative training were initiated on the 7th week post-stroke in rats with chronic, stable forelimb impairment. Despite the delay in starting therapy, VNS delivered with rehabilitative training produced significantly greater forelimb recovery compared to equivalent training without stimulation (Figure 1B). The degree of forelimb recovery after chronic stroke was comparable to that observed in previous studies of subacute stroke (Khodaparast et al., 2013, 2014). These findings provide an initial demonstration that the efficacy of VNS paired with rehabilitative training is not dependent on time to begin the intervention after stroke. Additionally, the observation that VNS therapy improves recovery when initiated long after stroke suggests that VNS does not act by augmenting the action of pro-plasticity factors upregulated in response to stroke (Khodaparast et al., 2016). Alternatively, VNS likely acts to enhance recovery by generating repeated, temporally precise, consistent engagement of pro-plasticity neuromodulatory circuits to reinforce rehabilitation-related neural activity (Hays et al., 2013; Hays, 2016). The independence from stroke-related plasticity is consistent with the ability of VNS paired with training to drive cortical plasticity in uninjured animals (Porter et al., 2012; Hulsey et al., 2016).

Advanced age is a major risk factor for stroke and is associated with reduced plasticity, which could in turn influence the effectiveness of VNS therapy. Thus, a study sought to determine whether VNS delivered during rehabilitative training could improve post-stroke recovery in aged rats (Hays et al., 2016). Rats aged at least 18 months were pretrained on a skilled forelimb task and subsequently underwent ischemic lesions of the motor cortex. Pairing VNS with rehabilitative training generated robust improvements in recovery of forelimb strength compared to equivalent training without VNS in aged rats. The magnitude of recovery observed in aged rats that received VNS therapy was comparable to that reported in previous studies using young rats receiving the same intervention (Khodaparast et al., 2013). The similar effectiveness in aged and young rats receiving VNS is consistent with studies suggesting that age alone is not a determinant in the benefits of rehabilitation and provides initial evidence that advanced age does not preclude VNS-dependent enhancement of post-stroke recovery (Bagg et al., 2002).

Generalization of improved functional recovery to tasks that are not explicitly trained during rehabilitation is an important consideration in the translation of therapies for clinical use, as it has practical implications for administration of the therapy. Given a fixed duration for a session of rehabilitation, a therapist would need to determine whether a patient should receive a greater number of stimulation pairings during a more constrained set of rehabilitative exercises or whether to deliver fewer stimulation pairings distributed across a greater breadth of rehabilitative exercises. To provide data to guide this determination, a recent study tested whether the VNS-dependent recovery after stroke would generalize to a similar, untrained task (Meyers E.C. et al., 2018). Rats were pre-trained on a task that measured skilled forelimb rotation, then underwent an ischemic lesion to motor cortex followed by training on the same rotational task with or without VNS. Delivery of VNS paired with rehabilitative training significantly enhanced recovery of forelimb rotation compared to equivalent training without VNS. After the completion of 6 weeks of motor training on the rotation task, all rats were tested on a similar, but distinct task that measured volitional forelimb strength. Rats that had previously received VNS paired with rehabilitative training on the rotation task exhibited significantly improved recovery on the volitional strength task compared to rats that had previously received rotation training without VNS, suggesting that VNS-dependent recovery may generalize to similar untrained movements. The magnitude of recovery observed on the untrained task was similar to that observed when VNS was paired with training on the primary task, providing evidence of generalization. Moreover, in this study, VNS-dependent recovery persisted at least 7 weeks following cessation of stimulation, providing additional corroborating evidence that the benefits of VNS are long-lasting.

Other studies provide insight into the implementations of VNS therapy that may be most beneficial. To determine the stimulation paradigm that yields the greatest enhancement in recovery, a study evaluated a range of distinct VNS parameters on recovery of forelimb strength after stroke (Hays et al., 2014b). Delivery of an equivalent amount of VNS that is temporally dissociated from rehabilitative training is less effective at promoting recovery than VNS that is paired with forelimb movement during rehabilitative training, suggesting that non-specific effects of stimulation that do not require precise timing, such as reduction of inflammation or neurogenesis, do not contribute to VNS-dependent enhancement of recovery. Additionally, a paradigm that delivered sixfold more stimulation in rapid succession generated significantly less recovery than VNS explicitly paired with forelimb movement rehabilitation. Together, the results from this study emphasize the need to optimize both the dose and timing of stimulation paradigms for VNS therapy.

**FIGURE 2:**
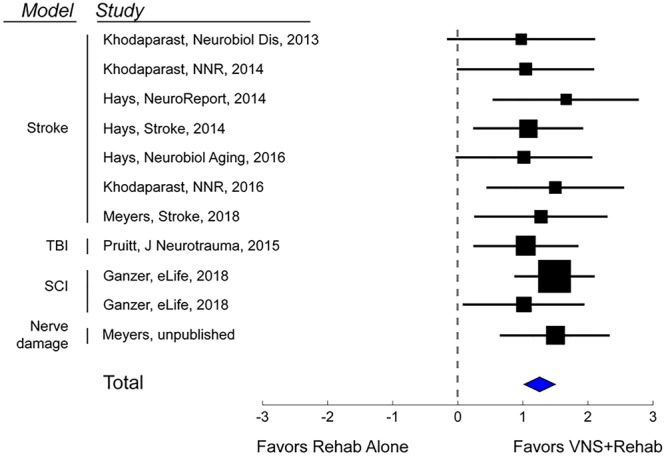
Vagus Nerve Stimulation therapy improves recovery in a variety of models of neurological injury. A meta-analysis of recovery across a range of rat models of neurological damage demonstrates that VNS paired with rehabilitative training (VNS+Rehab) consistently improves recovery of forelimb motor function compared to equivalent rehabilitative training without VNS (Rehab Alone). The data are presented as a forest plot. Markers denote standardized mean difference for VNS+Rehab compared to Rehab Alone for each study, and horizontal lines indicate 95% confidence interval. The size of the indicator represents the number of subjects. The blue diamond represents the summary effect.

Additional studies support the use of VNS therapy for mechanistically distinct forms of cerebrovascular injury. Intracerebral hemorrhage (ICH) is a common and devastating subtype of stroke with few post-injury treatment options. Evidence from preclinical studies indicates that reorganization of spared circuits supports recovery after ICH, similar to ischemic stroke (Auriat et al., 2010; Liang et al., 2013; Santos et al., 2013). Based on the premise that VNS enhances plasticity, a study evaluated whether VNS paired with rehabilitative training may lead to improved recovery in a model of ICH (Hays et al., 2014a). Rats were trained to proficiency on a skilled forelimb task and then received an injection of collagenase into the dorsolateral striatum to induce hemorrhage. Delivery of VNS paired with rehabilitative training significantly enhanced recovery compared to equivalent training without VNS, providing a preliminary demonstration that VNS therapy can improve motor function after ICH (Figure 1C). Emerging evidence extends these findings to other distinct forms of neurological damage, indicating VNS can improve recovery in models of traumatic brain injury (Pruitt et al., 2016), spinal cord injury (SCI; Ganzer et al., 2018), and peripheral nerve damage (Meyers E. et al., 2018; Figure 2).

Cognitive deficits are not uncommon in patients following ischemic stroke (Tatemichi et al., 1994). Preclinical studies document improvements in memory retention with VNS (Clark et al., 1995, 1998). While a small number of clinical studies provide corroborating evidence for the role of VNS in improving memory function, placebo-controlled studies in larger clinical populations are needed to determine whether VNS facilitates long-term improvement in cognitive function in humans after stroke (Hoppe et al., 2001; Boon et al., 2006; Ghacibeh et al., 2006; Sun et al., 2017). It is possible that short bursts of VNS combined with a cognitive rehabilitative training paradigm may promote plasticity and improve cognitive impairments after stroke. While considerably more development is needed, these findings raise the prospect that pairing VNS with cognitive rehabilitation may represent a potential intervention for post-stroke cognitive impairment.

Despite the evidence demonstrating VNS-dependent enhancement of recovery across a range of preclinical models of neurological injury, the mechanisms that underlie recovery are not thoroughly characterized. In the following section, we will discuss the putative mechanisms by which VNS modulates neural plasticity to support recovery of function.

## Neurobiological Mechanisms of Motor Recovery After Paired VNS

**FIGURE 3:**
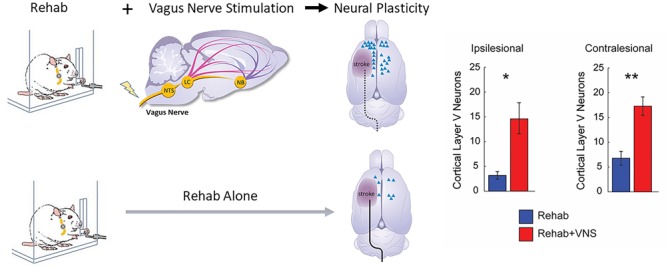
Vagus Nerve Stimulation paired with motor training enhances synaptic reorganization after stroke. Rats that receive VNS paired with motor training (red bar) after stroke demonstrated a significantly greater increase in corticospinal tract (CST) connectivity to rehabilitated muscles compared to equivalent training without VNS (blue bar). CST connectivity originating in both the ipsilesional and contralesional hemispheres was increased. These findings indicate that VNS drives large-scale reorganization in motor networks after stroke which may underlie recovery of function (Adapted from Meyers E.C. et al., 2018). ^∗^ indicates p < 0.05; ^∗∗^ indicates p < 0.01.

Structural plasticity in descending cortical spinal circuits has been associated with recovery after stroke. A recent study evaluated whether VNS paired with rehabilitative training influenced reorganization of corticospinal tract (CST) connectivity (Meyers E.C. et al., 2018). A retrograde transsynaptic tracing study in rats revealed that VNS paired with rehabilitation tripled synaptic connectivity in CST networks controlling the impaired forelimb compared to equivalent rehabilitation without VNS, providing a direct quantification of VNS-dependent plasticity in motor networks after stroke. This reorganization of CST connectivity was observed 2 months after the cessation of VNS, suggesting that this plasticity is robust and enduring, and consistent with the notion that this plasticity subserves long-term restoration of motor function (Figure 3).

Vagus Nerve Stimulation engages a variety of molecular and neuronal mechanisms via the ascending neuromodulatory systems that may underlie the observed reorganization of motor networks. After a stroke, treatment with brain-derived neurotrophic factor (BDNF) increases functional recovery, whereas reduction of BDNF levels prevented the benefits of rehabilitative training (Schabitz et al., 2004; Ploughman et al., 2009). In rodents, both acute and chronic VNS increased levels of BDNF in the hippocampus but the elevated BDNF levels were not associated with improvements in the forced swim or elevated plus-maze tests (Follesa et al., 2007). It remains to be determined whether elevated BDNF levels contribute to motor reorganization and stroke recovery.

Engagement of neuromodulatory networks that regulate synaptic plasticity also represents a means by which VNS likely supports recovery. VNS drives activation of multiple neuromodulatory networks, including the noradrenergic, cholinergic, and serotonergic systems (Nichols et al., 2011; Hulsey et al., 2017). These neuromodulators, in turn, act synergistically to alter spike-timing dependent plasticity (STDP) properties in active networks (Dan and Poo, 2004; Seol et al., 2007). These neuromodulators are known to act within a short window of approximately 5–10 s after neural activity, referred to as the synaptic eligibility trace, to allow STDP (He et al., 2015). Two studies provide initial evidence that VNS generates temporally precise neuromodulatory feedback within the synaptic eligibility trace to drive synaptic plasticity. First, in a study examining plasticity in auditory cortex, only tones presented concurrently with VNS were reinforced (Engineer et al., 2011). Tones delayed 15 s after VNS, which falls outside the time window for synaptic eligibility, failed to generate plasticity. Second, a study examined the requirement for a temporal association between VNS and optimal trials during rehabilitative exercises after SCI (Ganzer et al., 2018). VNS delivery immediately after or within 2 s of the optimal trials significantly enhanced recovery of motor function, while a delay of approximately 25 s from the optimal trials failed to yield any benefits compared to equivalent rehabilitation without VNS. These studies align well with the time scale of the synaptic eligibility trace and provide a means by which VNS may drive temporally precise neuromodulatory release to reinforce ongoing neural activity related to the paired event.

## Randomized Clinical Trials to Assess Safety and Efficacy of Paired VNS After Chronic Ischemic Stroke

Transitioning from basic science investigation to clinical studies moves the field closer to determining if these promising findings can translate into improvements in clinical care. Studies are now attempting to translate these preclinical VNS experiments into clinical practice through feasibility, safety, and more recently, pivotal clinical trials in individuals with chronic stroke (Dawson et al., 2016; Kimberley et al., 2018).

**FIGURE 4:**
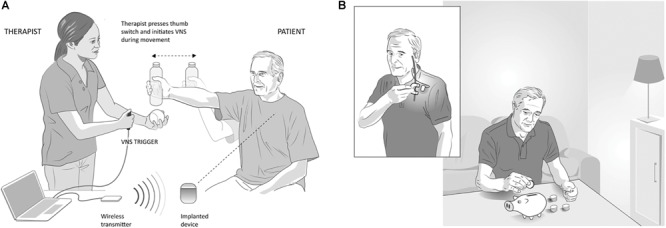
Vagus Nerve Stimulation paired with rehabilitation in the clinic and at home, **(A)** In-clinic rehabilitation with VNS: VNS is delivered by a therapist using a push button timed with a task-specific movement. Pressing the button delivers a brief burst of VNS (0.5 s) during an active goal-directed movement. The VNS system includes an implantable pulse generator (implanted device) that is implanted under the individual’s chest wall, an implantable lead, wireless transmitter (for communication between the device and computer) and custom programming software. **(B)** Home-based VNS therapy: Participants are provided a magnet (inset) to swipe over the device once before the start of each rehabilitation session to self-initiate 30 min of VNS (0.5 s burst of VNS every 10 s for 30 min). During the 30 min, participants performed at-home exercises prescribed by the therapist and adapted to their functional level and goals.

A single-blinded, randomized feasibility study evaluating VNS paired with motor rehabilitation was performed by Dawson et al. (2016) in 20 participants with chronic ischemic stroke who had moderate to severe upper limb weakness. Subjects were randomized to VNS paired with rehabilitation (*n* = 9; implanted) or rehabilitation alone (*n* = 11; not implanted). VNS was triggered by a therapist pushing a button during task-specific movements, based on the notion that VNS provides timed engagement of neuromodulatory networks to support rehabilitation-dependent plasticity (Figure 4A). Stimulation parameters were selected based on earlier preclinical studies (Engineer et al., 2011; Porter et al., 2012; Khodaparast et al., 2013, 2014, 2016; Hays et al., 2016; Hulsey et al., 2016). The main outcome measures were a change in upper extremity Fugl-Meyer Assessment (FMA-UE) score and response rate (FMA-UE change ≥6 points was considered clinically meaningful, discussed below). After 6 weeks of in-clinic rehabilitation, participants in the paired VNS group showed a 9.6-point improvement from baseline while the control group improved by 3 points in the per-protocol analysis (between group difference = 6.5 points, CI: 0.4 to 12.6, *p* = 0.038). The response rates were 66 and 36.4% in VNS and control groups, respectively. No serious adverse device effects were reported. These results demonstrated the feasibility of using paired VNS and did not raise safety concerns. Two limitations of this study were the absence of an implanted control VNS group and the lack of assessment of long-lasting effects of paired VNS. These limitations were addressed in a second pilot study (Kimberley et al., 2018).

This second study was a multicenter, fully blinded and randomized study (Kimberley et al., 2018). All participants were implanted with the VNS device, which allowed the control group to crossover to receive paired VNS therapy after completion of blinded follow-up and permitted within-subject comparison of gains. To evaluate the lasting effects of paired VNS, home-based therapy was included as part of the study (Figure 4B). Differences between the two studies are highlighted in Table 1.

**Table 1:** Comparison of the two pilot VNS studies (Dawson et al., 2016; Kimberley et al., 2018).

	Dawson et al., 2016	Kimberley et al., 2018
Number of sites	2 United Kingdom	4 United States and United Kingdom
Study design	Randomized, single-blind (Assessor)	Randomized, blinded (Assessor, Therapist, Participant), sham-controlled, cross-over
Number of participants	20 (VNS: *n* = 9; Control: *n* = 11)	17 (VNS: *n* = 8; Control: *n* = 9)
VNS implantation	Only VNS group implanted	Both VNS and Control group implanted
Long-term home therapy	No	Yes
Inclusion criteria	ARAT (Action Research Arm Test)	FMA-UE (Fugl-Meyer Assessment – Upper Extremity)
Outcome measure end-points	End of in-clinic (6 weeks) assessment followed by a 30-day assessment	End of in-clinic (6 weeks) assessment followed by 30-day and 3-month assessment
Imaging (Structural MRI)	Yes	Yes
Safety	(One) Transient vocal cord palsy and dysphagia after implant, (Five) minor events including nausea, metallic taste in the mouth. No serious adverse device effects.	(Three) Serious adverse events related to implantation surgery including wound infection, shortness of breath with dysphagia and hoarseness. No serious adverse device effects.
Efficacy (FMA change from baseline at 6 weeks)	9.6 vs. 3 (between group difference = 6.5 points, CI: 0.4 to 12.6, *p* = 0.038). ^∗^Response rate: 66% vs. 36.4%	7.6 vs. 5.3 (between group difference = 2.3 points, CI: -1.8 to 6.4, *p* = 0.2). ^∗^Response rate: 75% vs. 33%. At 3 months, post-therapy, 9.5 vs. 3.8 (between group difference = 5.7 points, CI: -1.4 to 11.5, *p* = 0.055). ^∗^Response rate: 88% vs. 33% (*p* = 0.03).

*^∗^FMA change ≥6 points.*

**FIGURE 5:**
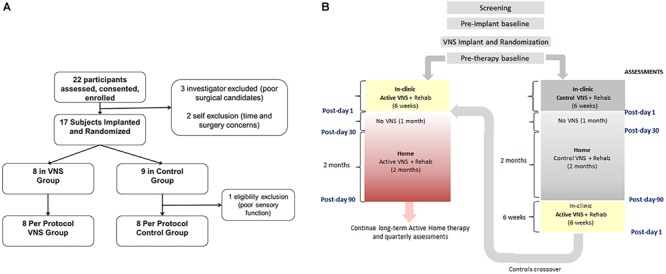
**(A)** Consort Diagram for the pilot study (from Kimberley et al., 2018). Twenty-two participants were enrolled in the study of which 17 were implanted. Eight participants were randomized to the VNS group and 9 to rehabilitation only **(B)**. Clinical study flowchart. After screening and baseline evaluations, all participants were implanted with a VNS device and randomized to receive either Active (0.8 mA) or Control VNS (0.0 mA) paired with upper limb rehabilitation. Participants received 18 sessions of in-clinic therapy for 6 weeks, followed by a home-based therapy for 3 months (no VNS was delivered to either group during the 1st month of home therapy). The 3-month time point is referred to as Post-day 90. After Post-day 90, the Active VNS group continued with home-based Active VNS, and the Control group crossed over to receive 6-weeks of in-clinic therapy with Active VNS followed by home-based Active VNS, similar to the Active VNS+Rehab group. Outcome measures were evaluated at baseline, Post-day 1, Post-day 30, and Post-day 90.

Seventeen participants with chronic ischemic stroke who had moderate to severe upper extremity impairment were enrolled at four sites, with similar surgical procedure and randomization (Figure 5A) to the first study. The study design is shown in Figure 5B. Participants performed 6 weeks of in-clinic therapy followed by home-based therapy. After 6-weeks of in-clinic therapy, participants in both groups had 1 month of at-home exercises with no VNS followed by 2 months of home-based therapy. During home therapy, participants in both groups activated the VNS device at the start of each 30-min session via a magnet swipe over the implanted pulse generator to deliver either Active or Paired VNS (0.8 mA) or Control VNS (0 mA), respectively (Figure 4B).

**FIGURE 6:**
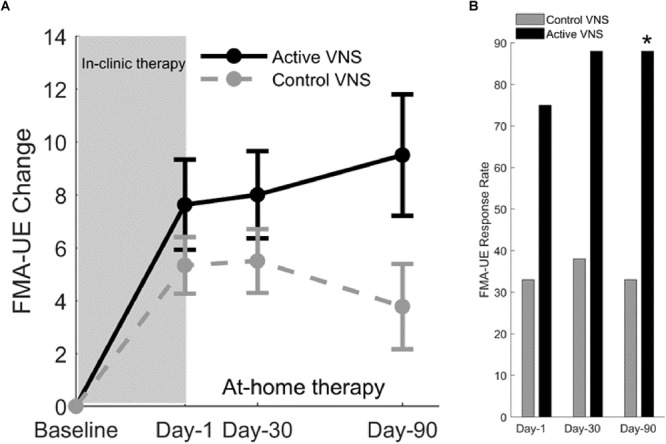
**(A)** Fugl-Meyer assessment–upper extremity (Kimberley et al., 2018). Change in FMA-UE score at three posttreatment assessments from baseline for Active VNS (solid line) and Control VNS + Rehab (dashed line). Shaded area indicates 6 weeks of in-clinic therapy. Error bars indicated standard error of the mean (s.e.m). **(B)** FMA-UE responder rate (defined as FMA-UE change ≥6 points from baseline) for Active VNS (black) and Control VNS + Rehab (gray). ^∗^*p* < 0.05, Fisher exact test.

After 2 months of home-based therapy, the Paired VNS group continued the VNS therapy while the Control Group switched over to receive paired VNS (Figure 5B). After 6 weeks of in-clinic therapy, the FMA-UE score increased by 7.6 points for the VNS group and 5.3 points for controls. Three months after the end of in-clinic therapy (post-90), the FMA-UE increased by 9.5 in the paired VNS group and 3.8 points in controls. At post-90, response rate (FMA-UE change ≥6 points) was 88% in the VNS group and 33% in controls (*p* = 0.03) (Figure 6A,B).

After controls crossed-over to receive in-clinic Active VNS, FMA-UE improved by 9.8 points from baseline (*p* < 0.001) after 6 weeks. After an additional 2 months of home-based VNS, FMA-UE improvement was maintained at 9.7 points (*p* = 0.01). Therefore, the improvements in upper limb impairment more than doubled after rehabilitation paired VNS compared to rehabilitation alone, an effect of approximately the same magnitude observed in the preclinical studies of VNS for ischemic stroke (Khodaparast et al., 2013).

It is of note that controls received similar intensity of in-clinic and home rehabilitation (without VNS) and showed minimal improvement in the randomized portion of the study, especially as more time elapsed following the in-clinic rehabilitation. After crossover to Active VNS, the control participants showed a clinically meaningful outcome that was similar to the initial Active VNS group. This is consistent with studies suggesting that intense rehabilitation or standard of care rehabilitation for individuals with chronic stroke may be insufficient to significantly improve motor outcomes (van der Lee et al., 1999; Langhorne et al., 2009; Teasell et al., 2014).

In addition to improving motor impairment, Active VNS therapy also improved upper limb functional performance. At post-90 (3 months after the end of in-clinic therapy), the Wolf Motor Function Test (WMFT-Functional) difference between the Paired VNS and Control groups was 0.33 points (CI, 0.04 to 0.61; *p* = 0.029). Thus, participants showed significant improvements on both impairment (FMA-UE) as well as functional scales (WMFT-Functional) after Paired VNS therapy. These results suggest that improvement reflects true motor recovery rather than improved movement compensation. The study showed that rehabilitation paired with VNS was an acceptably safe and feasible intervention for patients with chronic stroke and demonstrated sufficient safety and feasibility to support a larger pivotal trial.

The benefits of Paired VNS require time to emerge and may suggest that progressive neural reorganization is facilitated by paired VNS (Porter et al., 2012; Meyers E.C. et al., 2018). VNS responders had greater cortico-spinal tract (CST) injury compared to control responders, which suggests that VNS-induced neuroplastic mechanisms could facilitate improvements in the VNS responders who would otherwise not have responded to rehabilitation alone (Dawson et al., 2016). These findings also mirror the reorganization of the CST observed with VNS therapy in preclinical models in which VNS paired with rehabilitation significantly increased synaptic connectivity in both ipsilesional and contralesional CST networks controlling the impaired forelimb (Meyers E.C. et al., 2018; Figure 3). Assessment of plasticity in multiple brain regions that accompanies improvements in recovery would strengthen future clinical studies by providing a more detailed description of the mechanisms that support VNS-dependent benefits.

A change in FMA-UE score of ≥6 points was used to indicate a clinically meaningful improvement. Previous studies have assessed FMA-UE scores using anchor-based methods to determine the clinically important change in FMA-UE from baseline. The FMA-UE change ranged from 4.24 to 7.25 points (Page et al., 2012). A >50% improvement in the overall arm and hand function, which was considered an excellent improvement, corresponded to FMA-UE change of 5.25 points. If the 9.5-point increase in FMA-UE score observed at day-90 following Paired VNS and the 9.8-point change from baseline after crossover to Paired VNS in Controls is a true effect of VNS, the therapy enhances the modest improvements seen with rehabilitation alone, up to more clinically meaningful levels.

## Clinical and Neurophysiological Considerations for Future Clinical Studies

Although the studies described above present initial evidence that VNS paired with rehabilitation may support recovery after stroke, there are several important considerations for continued translation of the VNS therapy.

### Clinical and Neurophysiological Biomarkers

Clinical and neurophysiological biomarkers are important for predicting response to interventions, especially in a heterogeneous chronic stroke population (Milot and Cramer, 2008; Burke and Cramer, 2013; Wu et al., 2015; Boyd et al., 2017). It would be valuable to identify biomarkers in patient subpopulations that are non-responsive to the VNS therapy. Biomarker evaluation across a range of stimulation parameters, including intensity, frequency, and pulse width, would be useful to guide the selection of paradigms to maximize plasticity and recovery after stroke. Future studies with larger sample sizes may determine whether clinical and neurophysiological markers will help identify participants more or less responsive to VNS therapy.

A number of characteristics, including age, type of stroke (e.g., ischemic or hemorrhagic), stroke location (e.g., supratentorial or infratentorial), stroke severity, amount of spasticity, associated contractures that may limit movement, time since stroke onset, associated sensory loss, comorbidities (e.g., diabetes), are known to affect outcomes. Moreover, factors that directly impact neuromodulatory function, including Parkinson’s disease, Alzheimer’s disease and concomitant use of pharmacotherapeutic agents, may specifically impact the efficacy of VNS. These factors will be discussed below.

### Supratentorial and Infratentorial Strokes

The clinical VNS studies described above included participants with supratentorial, ischemic stroke and excluded infratentorial strokes. Infratentorial or posterior strokes such as those involving the cerebellum, pons or medulla, were excluded because the behavioral benefits of paired VNS have not yet been demonstrated in preclinical models. Furthermore, individuals with posterior strokes presenting with upper limb weakness likely have other symptoms including dizziness, double vision, visual field deficits, dysphagia, clumsiness of the hand and ataxia that may impact upper limb motor training and therefore would likely require a different rehabilitation protocol. Previous studies have demonstrated that brainstem infarcts can result in the activation or reorganization of motor cortex (Kwon and Jang, 2010). It is possible that Paired VNS therapy could recruit upstream spared CSTs to regain lost function. Furthermore, studies in rat models of SCI showed that VNS paired with motor training drives plasticity in upstream motor neurons, suggesting that VNS-dependent plasticity in residual cortical or subcortical motor circuits could mediate recovery (Ganzer et al., 2018).

### Hemorrhagic Stroke

In rat models of hemorrhagic stroke, rehabilitation improves motor outcomes along with changes in dendrite morphology suggesting that plasticity within residual neurons supported recovery (Auriat et al., 2010). Furthermore, studies in a rat model of ICH provide direct evidence that VNS paired with motor training significantly improves forelimb function compared to equivalent training alone (Hays et al., 2014a). However, the clinical VNS studies excluded individuals with hemorrhagic stroke to maximize the ability to detect effects in ischemic stroke patients. Considering the flexibility of VNS to enhance recovery in a wide range of neurological injury animal models including hemorrhagic stroke, future studies evaluating VNS in patients with these types of stroke is warranted.

### Age

Age is an important non-modifiable risk factor for ischemic stroke (Bagg et al., 2002; Kelly-Hayes et al., 2003; Saposnik et al., 2008; Hays et al., 2016; Lui and Nguyen, 2018). Advanced age is associated with a reduction in neuroplasticity, which raises the prospect that advanced age may reduce the efficacy of VNS therapy (Kelly-Hayes et al., 2003; Burke and Barnes, 2006; Freitas et al., 2011). However, preclinical studies provide an initial demonstration that age does not limit VNS-dependent enhancement of recovery after stroke, as aged rats benefited from the therapy as much as young rats (Hays et al., 2016). The pilot clinical study (Kimberley et al., 2018) included a wide age-range of participants (37–73 years), and after 3 months of paired VNS therapy, 50% of participants over 65 years of age showed significant improvement in FMA-UE scores (≥6-point change). Therefore, age by itself did not preclude VNS-dependent benefit in responders; and less improvement in non-responders suggests that other factors are involved in determining response to therapy.

### Chronic Stroke

The clinical studies included individuals with chronic stroke for the following reasons: First, highlighting the need for interventions that are effective long after the acute stroke episode, an estimated 7.2 million Americans live with chronic post-stroke disability (Benjamin et al., 2018). Second, evidence from preclinical studies supports the efficacy of VNS paired with rehabilitative training when initiated several weeks after stroke (Khodaparast et al., 2016). Thus, VNS likely acted by engaging plasticity-enhancing neuromodulatory circuits during training rather than pro-plasticity factors upregulated by stroke (Meyers E.C. et al., 2018). Third, since spontaneous recovery of upper limb motor deficits is often observed during the first 6 months after stroke, any improvements in upper limb deficits obtained from interventions carried out during this acute phase would be difficult to dissociate from this spontaneous recovery. Indeed, participants with sub-chronic stroke often show greater improvements on the FMA-UE compared to participants with chronic stroke (Shelton et al., 2001; Masiero et al., 2007; Narayan Arya et al., 2011). Finally, acute stroke is a life-changing event for the majority of individuals, and it is likely that most individuals, physicians, and other healthcare professionals would be somewhat reluctant to undergo a non-emergency surgical procedure. The chronic population was therefore selected as a starting point for investigation.

### Severity of Upper Limb Deficits

The VNS clinical studies excluded individuals with very severe upper limb deficits who had minimal to no movement in their upper extremity (typically FMA-UE < 15). VNS could be combined with other interventions to initiate movements in this severe population. Since paired VNS in rats facilitated recruitment of residual neurons and increased synaptic connectivity in cortico-spinal networks controlling the impaired forelimb (Meyers E.C. et al., 2018), it is possible that the severity of CST injury may not preclude recovery of the impaired limb function in humans. This would be an interesting area for study once proven effective in a moderately severe population.

### Centrally Acting Drugs May Interfere With the Effects of VNS

Since VNS acts via the activation of neuromodulatory pathways, it is possible that certain medications could interfere with the effects of VNS therapy. For example, lipophilic muscarinic antagonists (e.g., scopolamine) or adrenergic antagonists (e.g., metoprolol) easily cross the blood-brain barrier and are known to have central adverse effects which could interfere with the effects of VNS. Animal studies provide supporting evidence that interfering with neuromodulatory networks prevents the plasticity enhancing effects of VNS (Hulsey et al., 2016; Hulsey, 2018). Unlike pharmacological blockade, animal studies utilized methods that resulted in a permanent, virtually complete reduction of neuromodulators. Therefore, pharmacological antagonism may differentially influence the effects of VNS. Nevertheless, given the well-documented literature regarding the central effects of some cholinergic and noradrenergic antagonists on mood, cognitive processing, behavioral performance and neurophysiological indicators of plasticity, some drug exclusions need to be considered in clinical studies.

### Sensory Loss

Impaired tactile sensation, stereognosis, and proprioception are common after stroke. Sensory disruption can affect motor function and recovery, since sensorimotor integration is important for successful goal-directed movements (Xerri et al., 1998; Bolognini et al., 2016). With severe sensory loss, the motor deficits can appear to be worse, even in the absence of significant muscle weakness. The motor cortex receives significant input from somatosensory areas, and peripheral nerve lesions or lesions in the somatosensory cortex can significantly alter movement representations in motor cortex and impact motor skill learning (Donoghue and Sanes, 1987; Xerri et al., 1998). Furthermore, lesions of motor cortex can also disrupt sensory function (Nudo et al., 2000).

It is possible that repeatedly pairing VNS with tactile rehabilitation may improve sensory deficits in individuals with significant sensory loss. In a case report study involving a 72-year-old male with sensory deficits, VNS paired with tactile rehabilitation showed clinically meaningful improvements in sensory threshold, proprioception and stereognosis that were long-lasting (Kilgard et al., 2018). It is possible that the pairing narrowed receptive fields from the hand to individual fingers, which may have contributed to the improved tactile perception. Thus, individuals with motor deficits and significant sensory deficits may benefit from VNS combined with tactile training and could show improvements in both sensory as well as motor function.

### Comorbid Conditions

Neurodegenerative diseases (e.g., Alzheimer’s disease and Parkinson’s disease) can deplete neuromodulator reserves in basal forebrain cholinergic neurons and LC neurons (Whitehouse et al., 1981; Coyle et al., 1983; Gesi et al., 2000; Zarow et al., 2003). Since cholinergic and noradrenergic modulation is essential for the effects of VNS, it is possible that decreased neuromodulator reserves may impact VNS-induced plasticity. In such individuals, it is possible that different stimulation parameters may be needed to generate appropriate activation of remaining neuromodulatory networks. Future studies evaluating VNS in both animal models and patients with neurodegenerative diseases is warranted.

Future preclinical and clinical studies in larger populations along with neurophysiological biomarkers as predictors of improvement will help adapt the VNS therapy to different patient subgroups.

## Optimization of VNS Parameters

Identification of stimulation parameters and paradigms that yield maximal recovery is an important step in the translation of VNS-based targeted plasticity therapy for stroke. Both the preclinical and clinical studies evaluating motor recovery described above utilized identical stimulation settings of 0.8 mA, 100 μs pulse width, 30 Hz frequency and a pulse train of 0.5 s (Engineer et al., 2011; Porter et al., 2012; Dawson et al., 2016; Kimberley et al., 2018).

Given that VNS-directed plasticity is believed to underlie recovery, a number of studies have characterized stimulation paradigms aimed at increasing the magnitude of VNS-dependent plasticity. The parameter that has been most thoroughly investigated is stimulation intensity. Higher intensity stimulation recruits a larger proportion of vagal fibers and triggers stronger activation of neuromodulatory nuclei, which may improve stroke recovery (Roosevelt et al., 2006; Castoro et al., 2011; Mollet et al., 2013; Hulsey et al., 2017). Paradoxically, a number of studies examining the effects of VNS on neural plasticity and memory indicate that moderate intensity stimulation generates the greatest effects compared to lower and higher intensity stimulation (Clark et al., 1995, 1998, 1999), suggesting that non-linear interactions in upstream targets may be responsible for these effects and VNS operates across a specific range of stimulation parameters.

Increasing the pulse width can compensate for a reduction in stimulation amplitude, indicating that total charge delivered to the nerve is the main predictor of VNS-dependent engagement of neuromodulatory networks and VNS-dependent plasticity (Hulsey et al., 2017; Loerwald et al., 2017). Several studies have examined the influence of varying other stimulation parameters on VNS-dependent plasticity. Increasing the interval between stimulation trains increases the magnitude of VNS-dependent plasticity, an effect ascribed to desensitization of neuromodulatory receptors (Borland et al., 2018). Additionally, similar to the effect of stimulation intensity, the pulse frequency during a VNS train also demonstrates an inverted-U relationship with plasticity. Trains consisting of pulses delivered at moderate frequency rates enhanced cortical plasticity, while slower and faster pulse rates both fail to significantly enhance plasticity (Buell et al., 2018). Taken together, the studies illustrate the influence of both the timing and intensity of stimulation parameters on the magnitude of VNS-dependent plasticity, suggesting manipulation of either or both parameters may be required to optimize efficacy for clinical implementation.

The precise mechanisms that underlie the observed inverted-U relationship between plasticity and several VNS parameters are not fully understood. However, several possibilities could explain this response, the most apparent of which is the effect of stimulation intensity. First, lower stimulation intensities could recruit pro-plasticity neuromodulatory circuits, while higher intensities recruit overriding anti-plasticity networks. As a result, moderate stimulation intensities would produce the greatest enhancement of plasticity by maximally recruiting the low threshold system while suppressing activation of the high threshold system. Other possible explanations relate to receptor activation. Noradrenergic receptors are required for VNS effects and are known to display considerable adaptation (Gainetdinov et al., 2004). Low intensity stimulation may avoid desensitization and allow repeated effective signaling and thus drive plasticity, while high intensity stimulation may produce desensitization that prohibits repeated activation and limits plasticity. Alternatively, activation of different receptor types at differing stimulation intensities could produce an inverted-U effect. Low and moderate intensities of VNS may result in appropriate norepinephrine release to engage higher-affinity α2-adrenergic receptors and promote potentiation, whereas high intensity stimulation may increase norepinephrine levels further to activate lower-affinity β-adrenergic receptors to oppose potentiation. Indeed, this concentration-dependent dichotomy in control of the polarity of plasticity by adrenergic receptors has been described previously (Salgado et al., 2012). A recent study demonstrated that stimulation frequency also imposes an inverted-U effect on the degree of plasticity, consistent with postsynaptic receptor activation as the primary mediator of the response (Buell et al., 2018). It is important to note that both the desensitizing and opposing activation models, as well as many others, may contribute to the inverted-U, as they are not mutually exclusive.

It is not known whether the inverted-U response results from a common underlying principle of cellular and network activity across all brain regions or whether differences in network architecture across different systems would produce different outcomes. It is possible that non-responders to the standard VNS therapy may benefit from a different set of stimulation parameters that operate within this range or circumvent the conditions that perturb neuromodulatory pathways, such as alterations in vagal tone or neuromodulatory function. Furthermore, given the heterogeneity of patient characteristics as well as stroke manifestations described above, it is possible that some subgroups may be more responsive to one set of stimulation settings than others. Clinical studies described above utilized a standard, non-individualized set of stimulation parameters and observed significant improvement in motor deficits in most patients, supporting the notion that a relatively wide effective therapeutic range exists and individual variability is unlikely to preclude benefits (Dawson et al., 2016; Kimberley et al., 2018). Regardless of the underlying mechanism, the differential responses to stimulation parameters highlight the utility of optimizing stimulation parameters to yield the greatest response.

## Non-Invasive Vagus Nerve Stimulation

In recent years, non-invasive transcutaneous methods of stimulating the vagus nerve have emerged as a potential alternative strategy to generate VNS without necessitating a surgical implant. There are two primary ways of delivering non-invasive VNS. The first method, commonly termed tVNS or aVNS, targets the auricular branch of the vagus nerve (ABVN) and consists of the application of stimulation to the skin of the external ear on the tragus and cymba. The second is transcutaneous stimulation of the skin in the neck region over the cervical vagus nerve, commonly referred to as nVNS and targets the underlying cervical vagus.

The two main sites for auricular VNS include the tragus and cymba concha. Recent reports suggest that the extent to which vagal branches innervate the tragus is unclear (Badran et al., 2018a; Burger and Verkuil, 2018) due to inconsistencies in a human cadaver study that described the innervation of the human auricle (Peuker and Filler, 2002). Furthermore, inconsistencies in electrode placement and skin contact coupled with the effects of varying tissue impedance on nerve activation from individual to individual may be impediments to reliable stimulation with tVNS. For example, the electrode is placed over the auricular skin in a relatively small area with dense innervation and it is possible that the spread of current could activate nearby nerves such as the auriculotemporal branch of the mandibular nerve. This combined recruitment complicates the assessment and interpretation of the effects of stimulation of the vagus nerve.

Stimulation parameters using implanted cervical VNS have been well characterized and strongly influence the plasticity effects of VNS. The challenge of identifying and consistently delivering stimulation within a particular range of parameters is magnified by non-invasive stimulation strategies. While tVNS may be able to stimulate the auricular branches of the vagus, the inability to provide consistent, reliable activation may hamper the ability to observe robust effects. Furthermore, the ABVN has five times less A-β fibers compared to the cervical vagus nerve (Safi et al., 2016), which may contribute to its weaker activation of central targets (Ay et al., 2015).

Therefore, while avoiding surgical implantation has advantages, the preponderance of evidence in well-controlled studies points to the failure of these devices to sufficiently and reliably activate key brain structures. For example, in rat models of acute ischemic stroke, cervical VNS resulted in a greater reduction of infarct volume compared to non-invasive VNS (Ay et al., 2009, 2015). Non-invasive VNS also generated less intense c-fos staining in NTS neurons compared to cervical VNS, suggesting less robust activation (Ay et al., 2015). Available data from human studies describing regional brain activation in response to non-invasive VNS varies substantially from study to study (Kraus et al., 2007; Frangos et al., 2015; Yakunina et al., 2017; Badran et al., 2018a). Moreover, human studies using tVNS at the tragus failed to demonstrate significant activation of the locus coeruleus, a key brainstem nucleus in the actions of VNS, compared to sham stimulation (Yakunina et al., 2017; Badran et al., 2018b). These studies may explain the reduced efficacy of human studies with non-invasive VNS compared to cervical VNS (Bauer et al., 2016; Barbella et al., 2018).

A second non-invasive approach is stimulation delivered to the neck region above the cervical vagus nerve (nVNS). This method of non-invasive stimulation has shown efficacy for the treatment of acute episodes of cluster headaches and migraine (Silberstein et al., 2016; Goadsby et al., 2018; Grazzi et al., 2018; Tassorelli et al., 2018). The mechanism of action is thought to arise from VNS-driven activation of NTS, which in turn modulates the activity of the trigeminal cervical complex (TCC) (Moeller et al., 2018) and suppresses the transmission of nociceptive signals to higher pain processing centers (Bohotin et al., 2003). However, NTS also receives direct inputs from the trigeminal and cervical nerves. Since these nerves lie near the vagus nerve, it is possible that these nerves can also activate NTS via the spread of current. Indeed, trigeminal nerve stimulation or peripheral nerve stimulation can modulate nociceptive signals in the TCC via activation of NTS (Contreras et al., 1982; Lewis et al., 1987; Du and Zhou, 1990; Zerari-Mailly et al., 2005; Liu et al., 2014; Mercante et al., 2017) and have therefore been used for the treatment of headaches (Magis et al., 2007, 2013; Saper et al., 2011). Activation of these nerves during nVNS could contribute to headache relief (Henssen et al., 2019). Therefore, both VNS and TNS can modulate nociceptive input via NTS activation and may represent a generalized anti-nociceptive response to stimulation. In contrast, the induction of cortical plasticity is unique to VNS inputs. Repeatedly pairing a tone with cervical VNS, but not TNS, resulted in tone-specific plasticity in the auditory cortex (Engineer et al., 2011).

In addition to NTS, which receives 95% of the vagal input (Magdaleno-Madrigal et al., 2010), key brain regions activated by cervical VNS are also activated by non-invasive VNS including locus coeruleus, amygdala, hippocampus, cingulate and insula (Chae et al., 2003). This implied that the actions of non-invasive VNS were similar to cervical VNS since both methods activate similar upstream targets, and could, therefore, be used as an alternative to cervical VNS. However, many studies have demonstrated these key brain regions are also activated by peripheral nerve stimulation, trigeminal nerve stimulation, and cutaneous stimulation (Kwon et al., 2000; Rouzade-Dominguez et al., 2001; Scherder et al., 2003; Frangos and Komisaruk, 2017; De Cicco et al., 2018). Furthermore, LC neurons can be activated by both aversive stimulation (e.g., tail pinch) as well as cervical VNS (Hulsey et al., 2017). In other words, brain regions activated by VNS are also activated by tactile, arousing or aversive sensory stimuli, suggesting that the activation of these regions is not specific to the vagus nerve. Therefore, nVNS activation of common brain regions does not entail equivalence to cervical VNS.

Furthermore, cervical VNS stimulation parameters have been well characterized and have been shown to modulate plasticity effects across a twofold range of intensities and suggest the existence of a potentially useful therapeutic range of activity (Borland et al., 2016). With non-invasive VNS, the ability to deliver consistent and reliable stimulation within a particular range of parameters to induce plasticity for therapeutic use has not yet been demonstrated. Taken together, these results demonstrate that brain activation of common targets by cervical VNS and non-invasive VNS does not entail similar plasticity or behavioral outcomes. More studies are needed to determine the extent to which the vagus nerve is activated using non-invasive approaches along with a parametric characterization of stimulation parameters.

Recently, two clinical studies were conducted using non-invasive VNS combined with upper limb rehabilitation in individuals with chronic upper extremity weakness after stroke. In a study by Capone et al., (Capone et al., 2017) individuals with chronic ischemic or hemorrhagic stroke were randomized to either tVNS combined with robotic rehabilitation (*n* = 7) or auricular-sham VNS (ear lobe) combined with robotic rehabilitation (*n* = 5). The therapy was delivered for 10 days over 2 weeks. After 2 weeks, no significant differences between the tVNS and sham group were observed on the FMA-UE score (5.4 vs. 2.8 points, *p* = 0.16). While the results are interesting, the sample size precludes drawing distinct conclusions about tVNS efficacy.

In the second single-arm feasibility study (Redgrave et al., 2018), 13 participants more than 3 months post-stroke underwent rehabilitation combined with tVNS for 6 weeks. After tVNS rehabilitation training, the FMA-UE score increased by 17.1 ± 7.8 points with a >10-point change in 83% of patients. It should be noted that the FMA-UE scores used in this study combined motor, sensory, and joint components (0–126 points score) instead of the 0–66 points score that is typically used in many upper-limb stroke studies. Therefore the results are not directly comparable with the cervical VNS studies (Dawson et al., 2016; Kimberley et al., 2018). Several limitations of this study are worth considering. First, the study did not include a sham stimulation control group. Since stimulation was delivered at the maximally tolerable intensity and was thus perceptible, a placebo effect of stimulation cannot be ruled out. Second, some participants were less than 6 months post-stroke, and it is possible that spontaneous recovery could contribute to some of the improvement (Narayan Arya et al., 2011). A future randomized, blinded, placebo-controlled study in chronic stroke patients would be required to determine the efficacy of non-invasive VNS as applied to upper limb rehabilitation.

Further studies are needed to explore the effectiveness of non-invasive VNS, with a specific focus on parametric characterization. Ideally, any non-invasive VNS effects would be benchmarked against implanted VNS to determine the magnitude. As non-invasive stimulation would have demonstrable advantages for patients over implanted VNS, a thorough evaluation in robust, well-designed studies is needed to guide future clinical implementation.

## Conclusion and Future Directions

The studies reviewed provide a compelling demonstration that VNS-based rehabilitation is a potentially useful strategy to target plasticity and improve motor function for chronic stroke. VNS-dependent rapid engagement of neuromodulatory networks provides a signal to facilitate plasticity in pathways activated by rehabilitative exercises. While the effects of cholinergic and noradrenergic modulation on cortical plasticity have been well documented, other neuromodulators could also play a role in VNS-induced cortical plasticity. Emerging evidence highlights a similar role of serotoninergic systems in the VNS-dependent enhancement of plasticity, paralleling studies demonstrating that VNS activates these neuromodulatory systems (Manta et al., 2009, 2012; Hulsey, 2018). The neurophysiological mechanisms underlying VNS-driven cortical plasticity are complex and likely involve top-down control of neuromodulatory inputs involved in the planning of movements, reward, and decision making (Zmarowski et al., 2005; Convento et al., 2014).

The effects of VNS paired with rehabilitation have been tested across several different animal models of stroke and other neurological injuries and consistently demonstrate significantly greater recovery and enhancement of plasticity when rehabilitation is paired with VNS compared to equivalent rehabilitation without VNS. The flexibility to improve recovery across several injury models demonstrates that VNS engages a generalized mechanism to potentiate benefits specific to rehabilitation. The improved behavioral outcomes across different models along with objective evidence of plasticity after paired VNS informed clinical studies for the inclusion of appropriate patient populations who are likely to benefit from the therapy.

The encouraging findings from the two pilot clinical studies supported the design of a phase III pivotal, multi-site, double-blind, randomized trial (VNS-REHAB) of this intervention with 120 implanted participants and approximately 20 study sites. This study is powered to detect the difference seen in the FMA-UE score at the end of 6-weeks of in-clinic therapy with 80% power. The VNS-REHAB study is approximately 75% enrolled, with enrollment expected to complete in Spring 2019.

Despite the observed improvements across a range of conditions, it is possible that additional factors, including comorbid conditions, stroke etiology, individual variations in anatomy, and drugs or diseases that influence neuromodulatory function, could influence the efficacy of VNS therapy. Evaluation of the clinical effectiveness of paired VNS therapy in heterogeneous stroke populations along with continued development of stimulation parameters and rehabilitative paradigms to individualize and optimize the therapy for specific patient subgroups will improve the potential of this therapy to improve human function and well-being.

## Author Contributions

NE and SH wrote the manuscript. All authors participated in the discussion of the manuscript and provided significant revisions. All authors approved the final version of the manuscript for submission.

## Conflict of Interest Statement

NE, CP, and WBT are employees of MicroTransponder. JD and TK have presented some of this work at conferences and have received reimbursement. The remaining author declares that the research was conducted in the absence of any commercial or financial relationships that could be construed as a potential conflict of interest.
